# Supplementary material for: APPA Increases Lifespan and Stress Resistance via Lipid Metabolism and Insulin/IGF-1 Signal Pathway in Caenorhabditis elegans
Source: Int J Mol Sci. 2023 Sep 5;24(18):13682. doi: 10.3390/ijms241813682 (PMC10531162; doi:10.3390/ijms241813682)
Supplement: Supplementary file 1 [file ijms-24-13682-s001.zip › ijms-2576585-supplementary.pdf]

International Journal of *Molecular Sciences*

*Supplementary Materials*

# APPA Increases Lifespan and Stress Resistance via Lipid Metabolism and Insulin/IGF-1 Signal Pathway in *Caenorhabditis elegans*

**Shiyao Wang, Dongfa Lin, Jiaofei Cao and Liping Wang ***

Key Laboratory for Molecular Enzymology and Engineering of Ministry of Education, School of Life Sciences, Jilin University, Changchun 130012, China; shiyao21@mails.jlu.edu.cn (S.W.); lindf21@mails.jlu.edu.cn (D.L.); caojf1321@mails.jlu.edu.cn (J.C.)

* Correspondence: wanglp@jlu.edu.cn; Tel.: +86-431-8515-5348

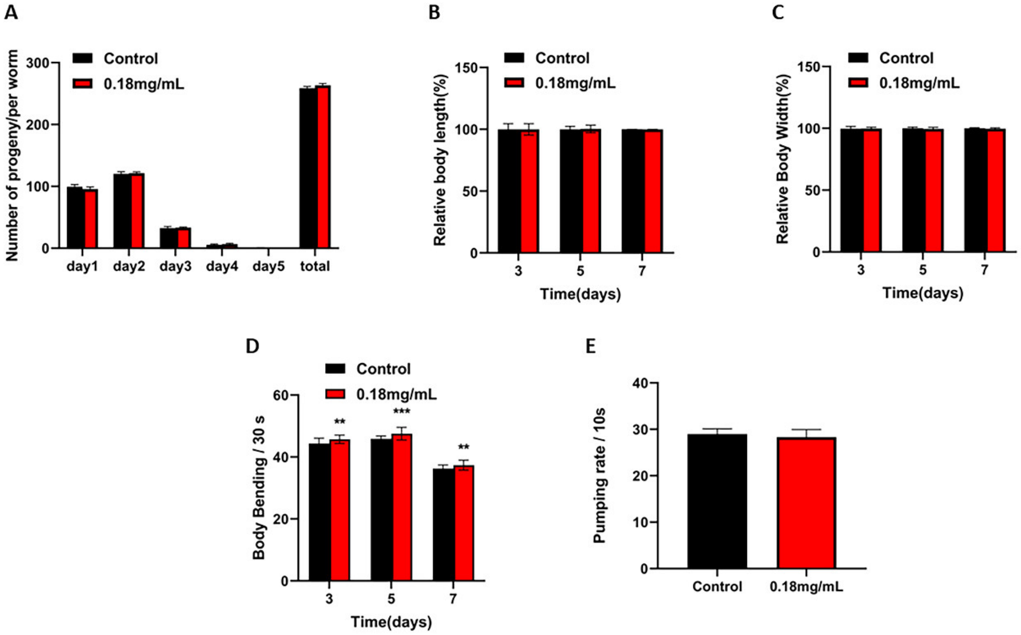


**Figure S1.** Effects of APPA on physiological indices of *C. elegans*, including spawning volume, body length, body width, bending frequency and swallowing frequency. (A) Egg production was counted daily from day 1 to day 5. Compared with the control group, APPA did not affect the reproductive capacity of nematodes. n=3(5 individuals per group) (B) APPA did not affect the body length of *C. elegans* compared with the control group. n=3(30 individuals per group) (C) APPA did not affect the body width of *C. elegans* compared with control group. n=3(30 individuals per group) (D) Compared with the control group, APPA enhanced the locomotor ability of *C. elegans*, especially on day 5. n=3(30 individuals per group) (E) Compared with the control group, APPA did not affect the food intake of nematodes. n=3(30 individuals per group).

**Citation:** Wang, S.; Lin, D.; Cao, J.; Wang, L. APPA Increases Lifespan and Stress Resistance via Lipid Metabolism and Insulin/IGF-1 Signal Pathway in *Caenorhabditis elegans*. *Int. J. Mol. Sci.* **2023**, *24*, x. https://doi.org/10.3390/xxxxx

Academic Editor: Andrew G. Ewing

Received: 9 August 2023
Revised: 31 August 2023
Accepted: 2 September 2023
Published: date

**Copyright:** © 2023 by the authors. Submitted for possible open access publication under the terms and conditions of the Creative Commons Attribution (CC BY) license (https://creativecommons.org/licenses/by/4.0/).

**Table S1 All strains used in this study.**

| Strains | Genotype |
| --- | --- |
| N2 | *C. elegans* wild isolate. |
| DA1116 | *eat-2*(ad1116) II. |
| CB1370 | *daf-2*(e1370) III. |
| GR1307 | *daf-16*(mgDf50) I. |
| EU1 | *skn-1*(zu67) IV/nT1 [unc-?(n754) let-?] (IV;V) |
| PS3551 | *hsf-1*(sy441) I. |
| CF1553 | muIs84 [(pAD76) *sod-3*p::GFP + rol-6(su1006)]. |
| MQ887 | *isp-1*(qm150) IV. |
| TJ356 | zIs356 [*daf-16*p::*daf-16*a/b::GFP + rol-6(su1006)] |
| TK22 | *mev-1*(kn1) III. |

**Table S2.** List of primers used for the quantitative real-time reverse transcription-polymerase chain reaction.

| Gene name | Primer sequence (5'to3') | Tm(°C) | bp |
| --- | --- | --- | --- |
| act-1F | CTACGAACTTCCTGAC-GGACAAG | 62 | 23 |
| act-1R | CCGGCGGACTCCATACC | 61.8 | 17 |
| sod-3F | CTCCAAGCACACTCTCCCAG | 61.9 | 20 |
| sod-3R | TCCCTTTCGAAACAGCCTCG | 59.9 | 20 |
| gst-4F | TGGCAAGAATGATGCCCTGT | 61.9 | 22 |
| gst-4R | CCAGCGAG-TCCAAATTTTCTTGCCA | 62 | 25 |
| hsf-1F | TTTGCATTTTCTCGTCTCTGTC | 56.3 | 22 |
| hsf-1R | TCTATTTCCAGCACACCTCGT | 58 | 21 |
| hsp-16.2F | GGTGCAGTTGCTTCGAATCTT | 58 | 21 |
| hsp-16.2R | TCTTCCTTGAAC-CGCTTCTTTC | 58.2 | 22 |
| daf-16F | CTAACTTCAA-GCCAATGCCACTA | 58.4 | 23 |
| daf-16R | TCCAGCTT-GACTCAGCTCATGTC | 52.2 | 23 |
| nhr-80 F | AATTCCGAT-TTCCAGCTTCTTC | 56.3 | 22 |
| nhr-80 R | TCTGCAGATTTGGTG-CATACTATAA | 57.1 | 25 |
| fat-6 F | GGCAAACCGTGAT-TTTCACATT | 56.3 | 22 |
| fat-6 R | TCACGAGCCCATTCGATGAC | 59.9 | 20 |
| skn-1F | AGAGAAATCGACAG-TAGCGAGAA | 58.4 | 23 |
| skn-1R | TCGGCTTTTTGAGTTGGATGT | 56.1 | 21 |

**Table S3.** The lifespan of N2 and mutant C. elegans.

| Genotype | Treatment | Mean | ±SD | Percent Change (%) | *p* value (log-rank significance) |
|---|---|---|---|---|---|
| N2 | 0 mg/mL | 20.9849 | 2.1984 | | |
| | 0.09 mg/mL | 21.7164 | 2.0163 | 3.49% | <0.0001**** |
| | 0.18 mg/mL | 22.0167 | 3.2119 | 4.92% | <0.0001**** |
| | 0.36 mg/mL | 21.5625 | 3.5054 | 2.75% | <0.0001**** |
| daf-2 | 0 mg/mL | 38.71 | 5.47 | | |
| | 0.18mg/mL | 39.29 | 5.65 | 1.49 | 0.8595 |
| daf-16 | 0 mg/mL | 14.41 | 2.40 | | |
| | 0.18mg/mL | 14.92 | 2.43 | 3.57 | 0.0650 |
| skn-1 | 0 mg/mL | 12.20 | 4.59 | | |
| | 0.18mg/mL | 12.28 | 4.52 | 0.69 | 0.8280 |
| hsf-1 | 0 mg/mL | 16.35 | 3.80 | | |
| | 0.18mg/mL | 17.14 | 4.16 | 4.85 | 0.1617 |
| eat-2 | 0 mg/mL | 20.13 | 8.44 | | |
| | 0.18mg/mL | 23.00 | 9.35 | 14.25 | 0.0227 |
| isp-1 | 0 mg/mL | 30.19 | 13.86 | | |
| | 0.18mg/mL | 30.47 | 13.55 | 0.93 | 0.8861 |
| mev-1 | 0 mg/mL | 17.09 | 5.24 | | |
| | 0.18mg/mL | 17.00 | 5.15 | -0.50 | 0.5196 |

**Table S4.** Top 10 genes upregulated by treatment with 0.18mg/mL APPA.

| Gene name | Description # | Overview * |
|---|---|---|
| F32D8.4 | PIH1 N-terminal domain-containing protein | Predicted to be involved in box C/D snoRNP assembly and rRNA processing. ribonucleoprotein complex. |
| C48B6.9 | Transmembrane protein | Predicted to be located in membrane. Predicted to be integral component of membrane. |
| F54D5.4 | DUF19 domain-containing protein | Involved in innate immune response. |
| C17E4.2 | Cilia- and flagella-associated protein 157 | Is expressed in head; intestine; and vulval muscle. |
| B0379.2 | RanBD1 domain-containing protein | Predicted to be located in membrane. Predicted to be integral component of membrane. |
| C04E12.4 | PAW domain-containing protein | Predicted to enable hydrolase activity. Predicted to be involved in glycoprotein catabolic process. |
| C16C8.18 | Tetratricopeptide repeat protein | Predicted to enable microtubule binding activity. Orthologous to human RMDN2 (regulator of mi- |

| | | |
|---|---|---|
| | | crotubule dynamics 2). |
| ZK596.2 | Protein kinase domain-containing protein | Predicted to enable protein serine/threonine kinase activity. Predicted to be involved in peptidyl-serine phosphorylation. |
| C09B9.2 | Transmembrane protein | Predicted to be integral component of membrane. |
| ZK354.6 | Protein kinase domain-containing protein | Predicted to enable protein serine/threonine kinase activity. |

# Data from NCBI (National Center for Biotechnology Information).
* Data from WormBase (https://wormbase.org/, accessed on, accessed on: 5, 29, 2023).

**Table S5.** Effects of different concentrations of APPA on N2 lifespan.

| **Genotype** | **Treatment** | **Mean lifespan (day) ±SD** | **Percentage change** | **p value (log-rank significance)** |
|---|---|---|---|---|
| N2 | 0 mg/mL | 20.9849±2.1984 | | |
| | 0.09 mg/mL | 21.7164±2.0163 | 3.49% | <0.0001**** |
| | 0.18 mg/mL | 22.0167±3.2119 | 4.92% | <0.0001**** |
| | 0.36 mg/mL | 21.5625±3.5054 | 2.75% | <0.0001**** |

**Table S6 The lifespan of N2 and mutant C.** elegans.

| Genotype | Treatment | Mean | ±SD | Percent Change (%) | p value (log-rank significance) |
|---|---|---|---|---|---|
| N2 | 0 mg/mL | 20.9849 | 2.1984 | | |
| | 0.09 mg/mL | 21.7164 | 2.0163 | 3.49% | <0.0001**** |
| | 0.18 mg/mL | 22.0167 | 3.2119 | 4.92% | <0.0001**** |
| | 0.36 mg/mL | 21.5625 | 3.5054 | 2.75% | <0.0001**** |
| daf-2 | 0 mg/mL | 38.71 | 5.47 | | |
| | 0.18mg/mL | 39.29 | 5.65 | 1.49 | 0.8595 |
| daf-16 | 0 mg/mL | 14.41 | 2.40 | | |
| | 0.18mg/mL | 14.92 | 2.43 | 3.57 | 0.0650 |
| skn-1 | 0 mg/mL | 12.20 | 4.59 | | |
| | 0.18mg/mL | 12.28 | 4.52 | 0.69 | 0.8280 |
| hsf-1 | 0 mg/mL | 16.35 | 3.80 | | |
| | 0.18mg/mL | 17.14 | 4.16 | 4.85 | 0.1617 |
| eat-2 | 0 mg/mL | 20.13 | 8.44 | | |
| | 0.18mg/mL | 23.00 | 9.35 | 14.25 | 0.0227 |
| isp-1 | 0 mg/mL | 30.19 | 13.86 | | |
| | 0.18mg/mL | 30.47 | 13.55 | 0.93 | 0.8861 |
| mev-1 | 0 mg/mL | 17.09 | 5.24 | | |
| | 0.18mg/mL | 17.00 | 5.15 | -0.50 | 0.5196 |

**RNA Sec**

## 1. RNA extraction

The animal total RNA was extracted according to the instruction manual of the TRlzol Reagent (Life technologies, California, USA).

## 2. Sample collection and preparation

### *2.1. RNA quantification and qualification*

RNA concentration and purity was measured using NanoDrop 2000(Thermo Fisher Scientific, Wilmington, DE). RNA integrity was assessed using the RNA Nano 6000 Assay Kit of the Agilent Bioanalyzer 2100 system (Agilent Technologies, CA, USA).

### *2.2. Library preparation for Transcriptome sequencing*

A total amount of 1 μg RNA per sample was used as input material for the RNA sample preparations. Sequencing libraries were generated using Hieff NGS Ultima Dual-mode mRNA Library Prep Kit for Illumina (Yeasen Biotechnology (Shanghai) Co., Ltd.) following manufacturer's recommendations and index codes were added to attribute sequences to each sample. Briefly, mRNA was purified from total RNA using poly-T oligo-attached magnetic beads.First strand cDNA was synthesized and second strand cDNA synthesis was subsequently performed. Remaining overhangs were converted into blunt ends via exonuclease/polymerase activities. After adenylation of 3′ ends of DNA fragments, NEBNext Adaptor with hairpin loop structure were ligated to prepare for hybridization. The library fragments were purified with AMPure XP system (Beckman Coulter, Beverly, USA). Then 3 μl USER Enzyme (NEB, USA) was used with size-selected, adaptor-ligated cDNA at 37°C for 15 min followed by 5 min at 95°C before PCR. Then PCR was performed with Phusion High-Fidelity DNA polymerase, Universal PCR primers and Index (X) Primer. At last, PCR products were purified (AMPure XP system) and library quality was assessed on the Agilent Bioanalyzer 2100 system.

### *2.3. Sequencing*

The libraries were sequenced on an Illumina NovaSeq platform to generate 150 bp paired-end reads, according to the manufacturer's instructions.

## 3. Data analysis

The raw reads were further processed with a bioinformatic pipelinetool, BMK-Cloud(www.biocloud.net) online platform.

### *3.1. Quality control*

Raw data (raw reads) of fastq format were firstly processed through in-house perl scripts. In this step, clean data(clean reads) were obtained by removing reads containing adapter, reads containing ploy-N and low quality reads from raw data. At the same time, Q20, Q30, GC-content and sequence duplication level of the clean data were calculated. All the downstream analyses were based on clean data with high quality.

### *3.2. Reads mapping to the reference genome*

The adaptor sequences and low-quality sequence reads were removed from the data sets. Raw sequences were transformed into clean reads after data processing. These clean reads were then mapped to the reference genome sequence. Only reads with a perfect match or one mismatch were further analyzed and annotated based on the reference genome. Hisat2 tools soft were used to map with reference genome.

### *3.3. Novel transcripts Prediction*

The StringTie Reference Annotation Based Transcript (RABT) assembly method was used to construct and identify both known and novel transcripts from Hisat2 alignment results

### *3.4. Gene functional annotation*

Gene function was annotated based on the following databases: Nr (NCBI non-redundant protein sequences);Pfam (Protein family); KOG/COG (Clusters of Orthologous Groups of proteins); Swiss-Prot (A manually annotated and reviewed protein sequence database); KO (KEGG Ortholog database); GO (Gene Ontology).

### *3.5. Quantification of gene expression levels*

Quantification of gene expression levelsGene expression levels were estimated by fragments per kilobase of transcript per million fragments mapped. The formula is shown as follow:

FPKM = {cDNA Fragments \over {Mapped Fragments(Millions) * TranscriptLength(kb)}} (1)

### *3.6. Differential expression analysis*

Differential expression analysis of two conditions/groups was performed using the DESeq2. DESeq2 provide statistical routines for determining differential expression in digital gene expression data using a model based on the negative binomial distribution. The resulting P values were adjusted using the Benjamini and Hochberg's approach for controlling the false discovery rate. Genes with an adjusted P-value < 0.01 & Fold Change≥2 found by DESeq2 were assigned as differentially expressed.

### *3.7. GO enrichment analysis*

Gene Ontology (GO) enrichment analysis of the differentially expressed genes (DEGs) was implemented by the clusterProfiler packages based Wallenius non-central

hyper-geometric distribution（Young et al, 2010）,which can adjust for gene length bias in DEGs.

*3.8. KEGG pathway enrichment analysis*

KEGG (Kanehisa et al., 2008) is a database resource for understanding high-level functions and utilities of the biological system, such as the cell, the organism and the ecosystem, from molecular-level information, especially large-scale molecular datasets generated by genome sequencing and other high-throughput experimental technologies (http://www.genome.jp/kegg/). We used KOBAS (Mao et al., 2005)database and cluster-Profiler software to test the statistical enrichment of differential expression genes in KEGG pathways.
